# Fine-grained time course of verb aspect processing

**DOI:** 10.1371/journal.pone.0264132

**Published:** 2022-02-25

**Authors:** Serge Minor, Natalia Mitrofanova, Gillian Ramchand

**Affiliations:** Department of Language and Culture, UiT - The Arctic University of Norway, Tromsø, Norway; Potsdam University, GERMANY

## Abstract

Sentence processing is known to be highly incremental. Speakers make incremental commitments as the sentence unfolds, dynamically updating their representations based on the smallest pieces of information from the incoming speech stream. Less is known about linguistic processing on the sub-word level, especially with regard to abstract grammatical information. This study employs the Visual World Paradigm to investigate the processing of grammatical aspect by Russian-speaking adults (*n* = 124). Aspectual information is encoded relatively early within the Russian verb which makes this an ideal testing ground to investigate the incrementality of grammatical processing on the sub-word level. Participants showed preference for pictures of ongoing events when they heard sentences involving Imperfective verbs, and for pictures of completed events when they heard sentences involving Perfective verbs. Crucially, the analysis of the participants’ eye-movements showed that they exhibited preference for the target picture already before they heard the end of the verb. Moreover, the latency of this effect depended on where the aspectual information was encoded within the verb. These results indicate that the processing and integration of grammatical aspect information can happen rapidly and incrementally on a fine-grained word-internal level. Methodologically, the study draws together a set of analytical techniques which can be fruitfully applied to the analysis of effect latencies in a wide range of studies within the Visual World eye-tracking paradigm.

## Introduction

Converging evidence from psycholinguistic research suggests that sentence processing happens in a highly incremental manner, and that the information from the incoming speech stream is rapidly integrated [1–6]. Less is known about linguistic processing on the sub-word level, especially with regard to abstract grammatical information. In this study we employed the Visual World Paradigm to investigate how quickly listeners are able to integrate grammatical information encoded within the word, focusing on the processing of grammatical aspect in Russian.

### Incrementality in sentence and word processing

In a seminal study, Tanenhaus et al. [6] showed that participants don’t wait until the end of temporarily ambiguous sentences to assign an interpretation, but make incremental commitments even at the risk of revisions at a later stage. It has also been shown that grammatical information, such as gender and case marking, is processed incrementally during sentence processing, which manifests itself in the ability of proficient speakers to use this information to anticipate certain properties of the upcoming speech signal (see [7–9] for recent overviews). For instance, grammatical gender information on agreeing elements (such as determiners and adjectives) may speed up the processing of a subsequent noun [10–14]. Similarly, Kamide et al. [15] in a Visual World eye-tracking experiment showed that comprehenders are able to rapidly integrate the information from grammatical case morphemes on nouns, and use it to predict a plausible continuation of the sentence. With respect to the timing of aspectual processing, Bott and colleagues [16–19] have investigated the increment size of aspectual interpretation. Bott and Gattnar [16] used the eyetracking-during-reading methodology to compare aspectual processing in German and Russian, and found evidence that aspectual information in Russian is integrated incrementally on a word-by-word level, as soon as the verb is processed.

While most previous studies have focused on word-by-word incrementality in sentence processing, a few have addressed the question of incremental processing on the sub-word level. In one of the first such studies, Allopenna et al. [20] employed Visual World eye-tracking to investigate the time course of lexical word recognition. They found that listeners generate hypotheses that are compatible with the minimally sufficient input—the onset of the word. Thus, upon hearing the initial syllable of the target word (e.g., ‘beaker’), participants launched significantly more gazes to the pictures of objects with onsets that matched the spoken input (in this case, ‘beetle’ and ‘beaker’) than to pictures of competitors which didn’t share the onset with the target noun (e.g., *speaker* and *carriage*). The interpretation was rapidly revised when more information became available, i.e., upon hearing the rhyme of the noun. Further support for incremental processing on the sub-word level has come from studies that analyzed event-related brain potentials (ERPs) to investigate the fine-grained time course of auditory speech processing. These studies found evidence that comprehenders make early attempts at semantic integration of lexical items with the context based on incomplete information about the perceived word [21, 22], and that word recognition involves incremental predictive processing on the sub-word level [23].

Thus, previous results suggest that the initial segments of words can be used predictively to anticipate word endings, and that the semantic integration of lexical information begins based on partial acoustic input. These findings have been taken as evidence for a *cascaded model of on-line language processing* [22, 24–26]. According to this model, no priority is given to any particular source or type of information during auditory word processing; instead all types of information (phonological, semantic, syntactic) are processed incrementally and in parallel, as soon as they become available in the input signal (immediacy principle, [26]). Moreover, even partial information obtained from bottom-up processing is rapidly integrated with knowledge from the linguistic and extra-linguistic context of the processed word (optimal use of contextual information, [24]).

Notably, existing studies of sub-word incrementality in semantic processing have focused on lexical contrasts involving words (mostly, nouns) that differ in their basic conceptual content (e.g., *beaker* vs *beatle* vs *speaker*). Much less is known about how highly abstract conceptual information associated with grammatical features is integrated on a fine-grained time scale below the word level. From a theoretical linguistic perspective, interpretable grammatical features (such as perfective and imperfective aspect) are commonly analyzed as encoding an abstract functional semantics that is ‘applied’ to the semantic content of the lexical word [27, 28]. In a similar vein, Bergen and Wheeler [29] have proposed that content words and grammatical features have a qualitatively different effect on the mental simulations triggered in the course of language processing. On this view, content words (e.g., *read, open*) provide the representations to be simulated, while grammatical categories (e.g., grammatical aspect) ‘operate over’ these representations, for instance by drawing focus to a particular part of the simulation (see also [30] for related observations). All this suggests the possibility that the semantic processing of grammatical features may be delayed as compared to the processing of lexical content, and in fact may have to occur subsequently to the processing of lexical content. Nevertheless, the cascaded model predicts that if interpretable grammatical features are manifested sufficiently early within the word, we should see evidence of early semantic integration of these features during auditory processing, even before the word is fully presented.

Our study was designed to test this prediction and fill the empirical gap with respect to the fine-grained time course of semantic integration of grammatical information. To do this we focused on the processing of grammatical aspect by adult Russian speakers. Verbal aspect in Russian is especially well-suited for the study of fine-grained incrementality of grammatical processing because a) aspectual information is morphologically encoded relatively early within the Russian verb, and b) previous studies have found robust effects of grammatical aspect on sentence processing using a range of behavioural measures. We address these points in turn.

### Grammatical aspect in Russian

Most verbs in Russian are marked as either Perfective or Imperfective (with the possible exception of the copula verb *byt’* ‘to be’, and a portion of so-called bi-aspectual verbs, which are commonly analysed as ambiguous between Perfective and Imperfective aspect). Perfective verbs are typically used to talk about events that were completed within a specific reference time period or immediately following a specific reference time point:

(1) Poka Petja rabota-l,       Sas̆a pro-c̆ita-l-a     knigu.
  While Peter work.imp-pst.m.sg Sasha pfv-read-pst-f.sg book
  ‘While Peter was working, Sasha read a book.’

This sentence entails that Sasha read the book to the end within the specified time period (i.e., while Peter was working). In this case, Perfective is used to mark a particular temporal relation between pairs of eventualities in a narrative sequence, specifically it signals that the event introduced by the Perfective verb is *fully contained* within the reference interval [28]. Perfective verbs are also commonly used to highlight the result of an event that holds at a certain reference point—typically, the speech time. In this use, Russian Perfective verbs are similar to Perfect constructions in other languages, e.g., in English.

On the other hand, Imperfective verbs are primarily used to refer to events that are ongoing at a given reference interval or time point:

(2) Poka Petja rabota-l,     Sas̆a c̆ita-l-a       knigu.
  While Peter work.imp-pst.m.sg Sasha read.imp-pst-f.sg book
  ‘While Peter was working, Sasha read a book.’

This sentence entails that the event of Sasha reading a book was ongoing during the specified time period. However, it does not entail that the reading event was completed during this period, i.e., this sentence is compatible with a situation where Sasha only managed to read half of the book during the period that Peter was working. Here, Imperfective aspect is used to signal that the relevant event *contains* the reference interval, without entailing the completeness of that event (see [31] on the importance of reference intervals expressed by *while*-clauses for the interpretation of imperfective aspect in Russian). Imperfective verbs are also used in habitual and Narrative Present contexts, and when referring to completed events without a definite reference point in the past (the so-called ‘general factual’ or ‘experiential’ reading, [32]).

Morphologically, non-derived verbs are predominantly Imperfective (although, non-derived Perfective verbs also exist). Perfective verbs can be derived from Imperfective verbs (and other Perfective verbs) via the addition of a prefix (compare the verb ‘read’ in ex. 1 and 2). Russian possesses a rich system of perfectivizing prefixes, and in many cases prefixation can significantly alter the lexical meaning of the verb (e.g., *c̆ita-* ‘read’ vs *ot-c̆ita-* ‘reprehend’, lit. ‘from-read’). In some cases, however, adding a prefix to an Imperfective verb has no or minimal impact on the basic lexical meaning, and only changes the verb’s aspectual properties (so-called *natural perfectives*, [33, 34]).

Conversely, Imperfective verbs can be derived from Perfective verbs via the addition of an imperfectivizing suffix (*-va, -yva, -a*) that precedes the tense/agreement inflection (these verbs are sometimes referred to as “secondary imperfectives”). This does not alter the lexical meaning of the verb.

In this study we focused on pairs of verbs that refer to the same type of event but differ in their aspectual specification (sometimes referred to as *actional pairs*, [35, 36], cf. also the notion of *aspectual pair*, [37–40]). Specifically, we were interested in pairs where the Imperfective verb can refer to an activity while the corresponding Perfective verb describes the natural completion (culmination) of that activity, i.e., an event where the activity reaches its natural endpoint/result. The verbs in these pairs share a common root morpheme, and the aspectual distinction between them is morphologically marked in one of two ways. In one class of pairs, the Imperfective verb lacks aspectual affixes, while the Perfective verb is derived from the Imperfective by the addition of a prefix. Here and below, the acute accent diacritic is used to mark the stressed vowel:

*c̆itá*-‘read (IMP)’   *pro-c̆itá*-‘read (PFV)’
  *strói*-‘build (IMP)’   *po-strói*-‘build (PFV)’

We will refer to such pairs as *prefixal pairs*.

In another class, both the Imperfective and the Perfective verbs include a prefix, and the Imperfective verb is derived from the Perfective by the addition of the imperfectivizing suffix. Often this is accompanied by an alternation in the root and/or stress shift affecting the root vowel:

*ras-krási*-‘color (PFV)’  *ras-krás̆i-va*-‘color (IMP)’
  *ot-krý*-‘open (PFV)’    *ot-kry-vá*-‘open (IMP)’

We will refer to such pairs as *suffixal pairs*.

Thus, in both types of pairs aspectual information is encoded relatively early within the verb. In prefixal pairs, it is signalled by the presence/absence of a (perfectivizing) prefix preceding the root. In suffixal pairs, it is encoded by the presence/absence of an (imperfectivizing) suffix immediately following the root, however in most cases the presence of the suffix can already be predicted based on the form of the root (weather the root vowel is stressed or not and/or the root has undergone an alternation). This makes Russian aspect an ideal testing ground to investigate the incrementality of grammatical processing on a sub-word level. Moreover, the (im)perfectivity of the verb (and hence reference to complete or ongoing situations) is determined earlier in verbs belonging to prefixal aspectual pairs than in verbs that belong to suffixal aspectual pairs. The existence of these two distinct types of aspectual marking (prefixal vs suffixal) makes it possible to test more subtle predictions with respect to the time-course of grammatical processing.

### Processing of grammatical aspect

Previous studies on the processing of grammatical aspect have confirmed the theoretical intuition that Imperfective aspect draws focus to the in-progress, activity stage of an event, while Perfective aspect triggers a representation of the event as a completed whole, highlighting the final stage and/or the result (goal) state of the event [30, 41–46] (for data on Slavic see [31, 47–51]). Madden and Zwaan [41] performed a series of experiments to test how grammatical aspect impacts the mental representation of events in English. In one experiment, participants were presented with sentences involving an accomplishment predicate, with the verb either in the Past Progressive (Imperfective) or the Simple Past (Perfective) form, e.g., *The man was making/made a fire*. They were then shown two pictures—one representing the event in an intermediate stage (e.g., a man bringing a lit match towards a pile of logs in the fireplace) and the other representing the event in a completed or near-completed stage (a man holding the match with the fire already lit). The participants were asked to choose the picture that best matched the sentence. Madden and Zwaan found that when participants read a sentence with a Perfective verb, they choose the end-stage picture significantly more often than the intermediate-stage picture. These results suggest that the Perfective aspect constrains the comprehenders’ representation of the event as completed.

Conversely, Imperfective aspect highlights the details of the activity stage of the event. This leads to a higher availability of features associated with the activity, including participants, instruments and locations [42, 52, 53]. Moreover, the use of Imperfective aspect to mark the event as in-progress increases the availability of the described event during subsequent narrative processing [54], with the contrast especially salient for telic (Accomplishment) predicates [55]. The claim that Imperfective aspect draws focus to the activity portion of the event is also supported by the fact that Imperfective verbs exhibit a congruency effect with the direction of motor movement, in contrast to Perfective verbs. This indicates that verbs in the Imperfective aspect can trigger a motor simulation of the described activity [29].

Zhou et al. [46] employed visual world eye-tracking to investigate the time course of grammatical aspect processing by Mandarin-speaking children (3, 4, and 5-year olds) and adults. The participants were shown two pictures: one representing an ongoing event (e.g., an old lady *planting* a flower) and the other representing the corresponding completed event (an old lady *having planted* a flower). While looking at the pictures, the participants heard sentences involving either a perfective or a durative (imperfective) morpheme that followed the verb. Zhou and colleagues found that all groups of participants launched significantly more looks to the completed event picture in the perfective condition, and conversely significantly more looks to the ongoing event picture in the durative condition. Moreover, the results showed that the participants reacted to aspect immediately after they heard the aspectual morpheme, before all of the verb’s arguments were introduced. These findings confirm that the processing of grammatical aspect information can proceed incrementally, at least on the word-by-word level. However, given that the aspectual morphemes in Mandarin Chinese always occur after the verb, these results cannot be used to answer the question of within-word incrementality in aspectual processing.

### The study

The design of our study was inspired by the paradigm in [46]. We used pictures of ongoing and completed events and eye-tracking to investigate the online processing of grammatical aspect in Russian. Crucially, the morphological properties of aspectual marking in Russian (i.e., the fact that aspectual marking is not word-final) made it possible to study the fine-grained time course of aspectual processing within the word.

We expected to replicate the previous behavioural finding that perfective verbs favour a completed event interpretation, while imperfective verbs favour an ongoing event interpretation. Our primary focus, however, was on the speed with which grammatical aspect information is integrated in the course of auditory processing. Our study addressed the following research question:

- RQ: Do adult speakers of Russian process and integrate grammatical aspect information as soon as it becomes available within the word?

In order to answer this question we wanted to test a) whether there is evidence that speakers of Russian integrate grammatical aspect information already before they reach the verb offset, and b) whether there is a detectable difference in the processing of aspectual information that is available earlier versus later within the verb, specifically whether aspectual information is processed earlier when it is encoded by the presence/absence of a prefix than when it is encoded by the presence/absence of a suffix.

## Materials and methods

### Participants

124 adult L1 speakers of Russian (age range 18–62, mean age = 22) participated in the experiment. The participants were recruited in Moscow among the students of Moscow State University and the Higher School of Economics, and the staff of two kindergartens. All participants were neurotypical adults capable of providing informed consent. They all provided consent prior to participation in the study in accordance with the Declaration of Helsinki. The Norwegian Centre for Research Data screened and approved the experiment. The participants read an information sheet that gave details of the experimental procedure, confidentiality and the right to withdraw at any point during the experiment. The participants’ data were anonymized, and their personal data were not stored after the testing phase was complete. All participants received a bookstore gift card for approximately $8 as compensation. All participants had normal or corrected-to-normal vision.

### Materials

**Fig 1:**
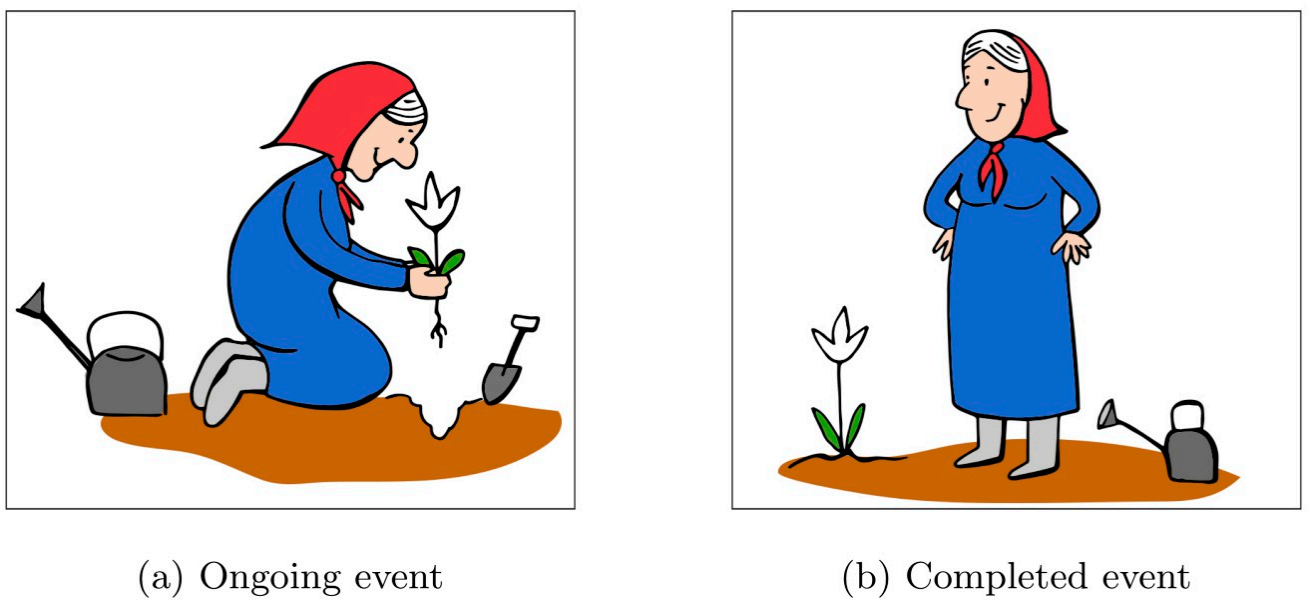
Visual display: ‘Grandma planting a flower’.

Experimental stimuli consisted of 24 test items, each including a visual display, a spoken preamble sentence and a spoken target sentence. All the target sentences involved a verb in the past tense, and we manipulated only the grammatical aspect of the verb (perfective or imperfective). A participant only heard either the perfective or the imperfective version of each target sentence (see S1 Appendix for a full list of target sentences). Each visual display contained two pictures located side by side representing different stages of the same event type: one representing an ongoing event, i.e., an action in progress (Fig 1a) and one representing a completed event, i.e., the result that obtained after the action was completed (Fig 1b).

The preamble sentences involved a short description of a scene in past tense, and were used to locate the narrative at a specific point in the past (e.g., ‘*It was raining*’, ‘*It was a sunny day*’, see ex. 3). The use of the preamble allowed us to restrict Perfective and Imperfective verbs to their narrative readings. For instance, the introduction of a specific reference situation in the past allowed us to rule out the so-called ‘general factual’ reading of imperfective verbs in Russian [32]. This was important, since on the general factual reading imperfective verbs can be used to refer to completed events.

All target sentences had the following structure: Subject—Transitive Verb—Object. The subject referred to one of four characters: grandpa, grandma, a boy, or a girl. The object always involved a combination of an adjective and an inanimate noun. A standardized 350 ms pause was inserted between the verb and the object. The two variants of the target sentence in each item involved verbs with the same lexical content, but differing in grammatical aspect: one perfective and one imperfective (cf. ex. 4a, 4b).

(3) Byl   soln^*j*^ec̆nyj den^*j*^.
  be.pst sunny  day
  ‘It was a sunny day.’
(4)
  a Babus̆ka saz̆a-la     b^*j*^elyj cv^*j*^etok.
    grandma plant.imp-pst white flower
    ‘Grandma was planting a white flower.’
  b Babus̆ka posadi-la  b^*j*^elyj cv^*j*^etok.
    grandma plant.pfv-pst white flower
    ‘Grandma planted a white flower.’

In half of the items, the imperfective target sentence involved an un-prefixed imperfective verb and the perfective target sentence involved the corresponding perfective verb derived from the imperfective by the addition of a prefix (prefixal aspectual pairs). In the other half of the items, the perfective target sentence involved a prefixed perfective verb and the imperfective target sentence involved the corresponding imperfective verb derived from the perfective by the addition of the imperfectivizing suffix (suffixal aspectual pairs). In ten out of twelve suffixal items, the addition of the imperfectivizing suffix was accompanied by a root alternation and/or stress shift affecting the root vowel. The lemma frequencies of the verbs in the prefixal and suffixal aspectual pairs were matched using Lyashevskaya and Sharov’s Russian frequency dictionary [56] based on the Russian National Corpus (approx. 92 million words). The mean log transformed frequencies of the verbs in the prefixal pairs (mean raw frequency = 28.48 i.p.m.; mean log transformed frequency = 1.1) and the suffixal pairs (mean raw frequency = 26.63 i.p.m.; mean log transformed frequency = 0.89) did not differ significantly (t = 1.12, p = 0.27). We further conducted a norming study to ensure that the visual displays matched the target sentences equally well for the prefixal and suffixal aspectual items (see below).

All audio stimuli were recorded by a female voice artist, who is a native speaker of Russian. The audio stimuli were recorded in a professional sound-proof studio. The average length of the perfective and imperfective verbs in the test items was closely matched (725 and 736 ms, respectively). Crucially, the average duration of the verbs in our study (731 ms) was long enough that if participants did react to early aspectual cues within the verb (e.g., in the prefix or in the root) we would expect to see an effect already before the verb offset.

Each participant was randomly assigned to one of four lists. In each list each target item occurred once, with either a perfective or an imperfective target sentence. Across the four lists, each item occurred twice with a perfective target sentence and twice with an imperfective one. The position of the ongoing/completed event pictures and the target pictures on the screen was counterbalanced across the four lists.

In addition, 24 filler items were included in the experiment. In each filler item, the visual display involved two pictures representing different event types. The preambles and the target sentences in the fillers were similar to those in the test items. Half of the filler items involved an imperfective verb, and half involved a perfective verb. However, since the pictures in the filler items represented different event types, the target picture could be identified solely on the basis of the lexical meaning of the verb (see S2 Appendix for a full list of filler sentences). In total, the experiment included 48 trials (24 test items and 24 fillers).

### Norming study

In addition to the main experiment, we conducted a norming study to evaluate how well the target sentences matched the visual displays. 46 adult Russian speakers participating in the norming study. The study was conducted online, and the participants were paid a small sum for participation. On each trial the participants were presented with a visual display that involved two pictures located side by side on the screen, followed by two sentences describing the pictures. The participants were asked to rate how well the sentences described the pictures on a scale from 1 (very bad) to 5 (very good). The study included 24 test items that involved visual displays and target sentences corresponding to the test items of the main experiment, and 24 filler trials corresponding to the filler items of the main experiment. In addition, 12 negative response items were included where the target sentences did not match the pictures of the visual display. These items involved pictures and sentences that were not part of the main experiment, and served as filters to control for participants’ attention and to encourage the use of low scores. In total the study included 60 trials, and lasted approximately 15–20 minutes.

Eight participants who gave scores higher than 2 for at least two negative response items were excluded prior to analysis. For the remaining participants (n = 38), the mean score of the test items was 4.8, with all test items scoring above 4.6. The mean scores for the prefixal and suffixal items were very closely matched (4.8 and 4.79 respectively).

### Procedure

The eye-tracking experiment was conducted in a lab setting. Participants were seated in a chair in front of a 22” monitor, and visual stimuli were displayed on the screen of the monitor. Eye movements were recorded by an SMI RED500 eye-tracker attached to the bottom of the monitor sampling at 120 Hz. Participants were seated at a distance of approximately 60–70 cm from the eye-tracker. Audio stimuli were presented via speakers located on both sides of the monitor.

Before the experiment, the participants were told that they would hear a series of short stories, and that at some point two pictures would appear on the screen. The participants were asked to point to the picture that best matched the story that they had heard. After the instructions, the calibration and validation routine was carried out.

**Fig 2:**
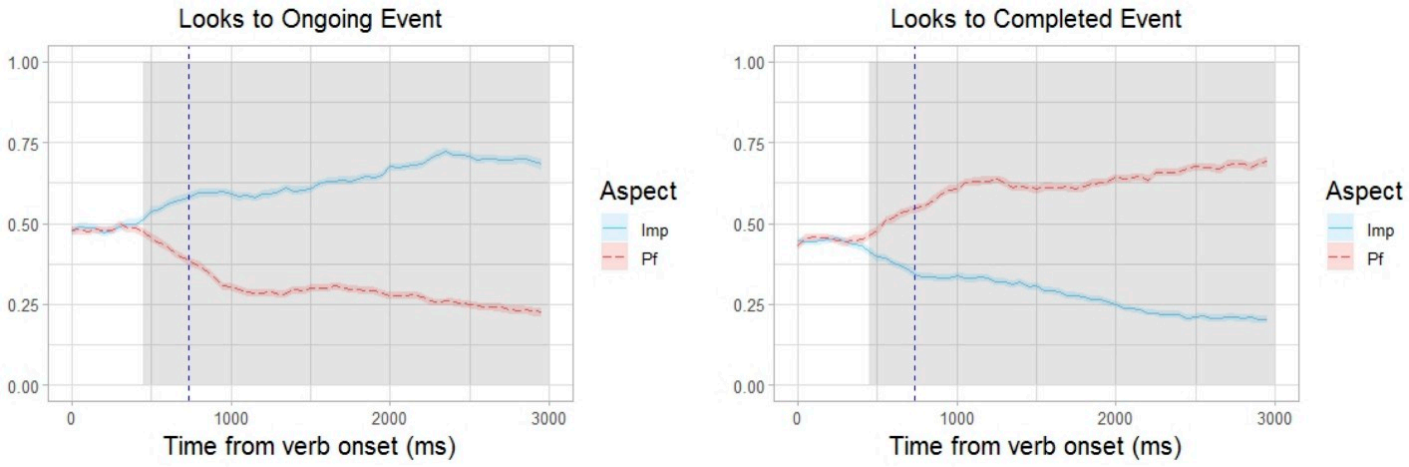
Looks to ongoing event and completed event pictures. Proportions of looks to the Ongoing Event (left panel) and Completed Event (right panel) pictures in 50 ms time bins starting from verb onset by condition (Imperfective vs Perfective). The vertical dashed blue lines represent the average verb offset. Shaded areas represent significant clusters identified via a cluster-based permutation analysis.

To familiarise the participants with the procedure, the experiment began with a filler trial. Each trial consisted of two stages: the preamble stage and the target stage. At the preamble stage, the participants were presented with a small picture of a smiley face in the middle of the screen and heard an audio recording of the preamble sentence. After the preamble sentence was presented the trial proceeded to the target stage. Two pictures representing different stages (ongoing vs completed) of the same event (in the test items) or different types of events (in the filler items) were displayed side by side on the screen (see Figs 1 and 2). After a pause of 500 ms, the participants were presented with an audio recording of the target sentence. The participants’ task was to select the picture on the screen that best matched the situation described by the target sentence. The participants selected a picture by lifting the left or the right hand (to select the left or the right picture, respectively). After a response was given, the experiment proceeded to the next trial. The participants’ eye-movements during the whole trial were recorded. Each experimental session lasted approx. 6 minutes.

### Predictions

Since the semantics of perfective aspect in Russian involves the notion of completion, we predicted that the participants would show preference for the picture of the Completed event when they heard a target sentence involving a perfective verb, reflected in more looks to the Completed event picture in the time period following the presentation of the verb. Conversely, since the imperfective aspect in the narrative context is used to talk about events in progress without the implication of completeness, we expected the participants to show online preference for the Ongoing event picture when they heard a sentence with an imperfective verb.

As discussed above, aspectual information is available relatively early within the verb in Russian: preceding the root in prefixal aspectual pairs, and at the root or immediately following the root in suffixal aspectual pair. This means that if the integration of aspectual information happens incrementally as soon as it becomes available, we expected that the participants would exhibit online preference for the target picture already before they heard the end of the verb.

Finally, if the processing of aspectual information is incremental on a fine-grained, word-internal level, we expected the time-course of aspectual processing to be different depending on where within the verb the aspectual information is encoded. Specifically, we expected the participants to show online preference for the target picture earlier when hearing verbs where the aspectual information is encoded by the presence/absence of a prefix (prefixal aspectual pairs), than for verbs where this information is encoded by the presence/absence of a suffix (suffixal aspectual pairs).

### Statistical analyses

To assess the participants’ eye-movement patterns, we calculated the proportions of looks to the Ongoing and Completed event pictures in 60 consecutive 50 ms time bins starting from the onset of the verb. To analyse the overall effect of Aspect on the gaze patterns, we conducted a *cluster-based permutation analysis* [57]; see also [58–60] for applications of this method in Visual World eye-tracking. This method is preferable to applying mixed models or ANOVAs to aggregated data or performing growth curve analysis [61] because: a) it provides correction for multiple comparisons without sacrificing statistical power, b) does not lead to an inflated Type 1 error rate due to autocorrelation, and c) allows localization of the effect in time without relying on an arbitrary selection of time windows [60, 62]. The analysis was conducted separately to evaluate the effect of Aspect on the looks to the Ongoing event picture and the looks to the Completed event picture. Trials with over 50% track loss were excluded prior to the analysis. This resulted in the exclusion of 7 trials (0.2% of the data). The analysis involved the following steps. First, we binarized the data in each time bin: if the proportion of looks to the critical picture was lower than 0,5 the time bin was assigned 0, otherwise 1. This affected only 5.3% of the time bins in the analysis of looks to the Ongoing Event picture (i.e., in 94.7% of the time bins the proportion of looks was already 0 or 1 prior to binarization), and 5.4% of the time bins in the analysis of looks to the Completed event picture. Next, we fit a mixed-effects logistic regression which evaluated the log-odds of looks to the critical picture as predicted by the Aspect of the verb in the test sentence. Participants and items were included as random intercepts. The model was run on each 50 ms time bin starting from the Verb onset up to 3000 ms after the Verb onset. Clusters were identified based on adjacent time bins where the p-value was below 0.05. The *z*-values for all the time bins within a cluster were summed up to generate the sum statistic for the cluster. Next, we randomly permuted the aspectual condition labels (Perfective vs Imperfective): for each participant the condition labels were permuted in either all or none of the trials with 0.5 probability. We then ran the model to identify significant clusters of time bins for the permuted data. We calculated the sum-statistic for each cluster, and the largest sum-statistic was stored. This process (permutation of the data and calculation of the largest sum-statistic) was performed 1000 times, generating a distribution of sum-statistics under the null hypothesis that Aspect does not have an effect. It was then possible to assess the sum-statistics obtained for the clusters in the original dataset against the null-hypothesis distribution, and obtain p-values for these clusters.

The cluster-based permutation analysis gives information about the significance and temporal localization of an effect, i.e., it tells us that a statistically significant effect obtains within a particular time range. However, it does not provide a reliable estimation of the onset latency of the effect, with a risk of both over- and underestimation [63]. To assess the fine-grained time-course of aspectual processing, we analysed the probability of looks to the Target picture at 150 time points starting from 200 ms after the Verb onset. The eye-movements were sampled at 120 Hz, which means the data contained one data point approx. every 8.33 ms. We used the *lme4* package in R to fit a mixed effects logistic regression predicting the log-odds of looks to the Target picture at each time point. Participants and Items were included as random intercepts. Only data involving looks to the Target and Competitor pictures were included in the analysis (5.4% of the data excluded). We used the Holm-Bonferroni method to correct the results of multiple comparisons carried out at individual time points. This generated a set of time points where the probability of looks to the Target picture was significantly above chance (*α* = 0.05) while controlling the family-wise error rate. The earliest of these time points was taken as the *upper estimate of the effect latency*, i.e., given that we employed a strong control for the family-wise error rate [57], we can be confident that the actual onset of the effect either coincided or preceded this time point.

Next, to investigate whether the time course of grammatical aspect processing was different for aspectual pairs involving Prefixal vs Suffixal aspectual marking, we compared the probability of looks to the target picture in trials involving these two classes of items. We ran a mixed effects logistic regression predicting the log-odds of looks to Target at 150 time points starting from 200 ms after the Verb onset, separately for the trials involving Prefixal and Suffixal items. The estimate of effect latency was taken to be the first of five consecutive time points with above-chance looks to the Target picture [64, 65]. To determine whether the difference in effect latencies between the conditions was statistically significant, we conducted a *bootstrapping divergence point analysis* (BDP, [66]; see also [67, 68]). The advantage of this analysis is that it is able to provide an estimate of the uncertainty around the difference in effect latencies between two conditions. The analysis involved re-sampling the data with replacement 3000 times, which was performed by trial within subjects, aspect labels (Perfective vs Imperfective) and item types (Prefixal vs Suffixal). For each re-sampled dataset, we calculated the estimates of effect latency for the Prefixal and Suffixal items, and the difference between them. The estimates were determined in the same way as for the original dataset. After 3000 iterations, we obtained bootstrapped distributions of the effect latencies for the Prefixal and Suffixal items, and of the difference between these latencies. Finally, we used the percentile method to obtain 95% confidence interval for the difference in effect latencies. Note, that this type of analysis presumes the presence of an effect to provide an estimate of effect latencies and to compare effect latencies between conditions. However, it may not be appropriate to test whether an effect exists in the first place and does not provide a statistical measure of the effect size [66, 69]. Thus, this analysis is complementary to the cluster-based permutation analysis described above.

To further verify the results of the BDP analysis, we applied a *bootstrap hypothesis testing procedure* (BHT) to directly test the null hypothesis that the latencies in Prefixal and Suffixal trials were not different, against the alternative hypothesis that the effect latency in trials involving Suffixal items was longer than in trials involving Prefixal items [70–72]. This procedure involves estimating the bootstrap distribution of the relevant statistic (in our case, the difference between the estimates of effect latency in Suffixal and Prefixal trials) under the null hypothesis, and then determining the probability of the actual value of the statistic relative to this distribution. The bootstrap distribution under the null hypothesis was obtained by randomly permuting the condition labels (Prefixal vs Suffixal aspect coding) on each iteration of the bootstrap. Label permutation was performed by trial within subjects and aspect labels. This bootstrap + permutation procedure was performed 3000 times, generating 3000 values for the difference in effect latency under the null hypothesis that the type of aspectual marking (Prefixal vs Suffixal) does not have an effect. The probability of the observed test statistic under the null hypothesis (p-value) was identified as the proportion of bootstrapped test statistics with values equal or greater than the observed value.

Finally, to make sure that our conclusions are not dependent on one particular measure of effect latency, we conducted the BDP and BHT analyses on the upper estimates of effect latencies for the Prefixal and Suffixal items, which were taken as the earliest time points with a significant effect after the application of the Holm-Bonferroni correction for multiple comparisons.

## Results

### Effect of aspect

First of all, we were interested in whether the participants would show different offline and online preferences for the Ongoing and Completed Event pictures depending on the grammatical aspect of the verb in the test sentence. In their offline responses, the participants showed an at-ceiling (> 95%) preference for the Ongoing event picture in the Imperfective condition, and conversely the Completed Event picture in the Perfective condition.

**Fig 3:**
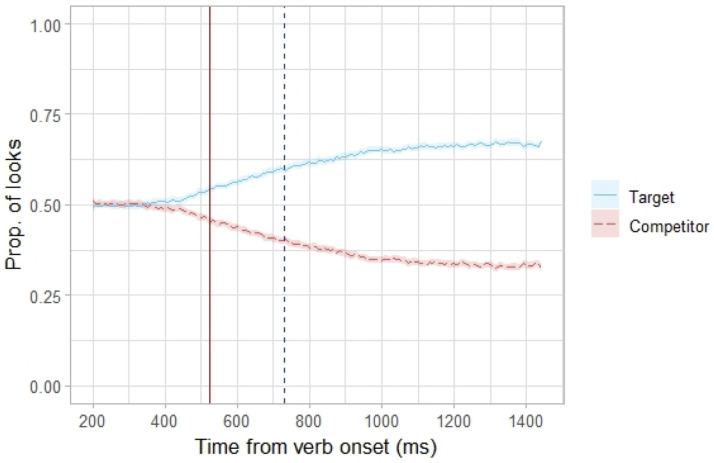
Looks to target and competitor pictures. Proportion of looks to the Target and Competitor pictures at 150 time points sampled at 120Hz starting from 200 ms after the verb onset. The vertical dashed blue line represents the average verb offset (731 ms). The vertical solid red line represents the upper estimate of effect latency (525 ms), i.e., the earliest time point with above chance looks to the Target picture after applying the Holm-Bonferroni correction for multiple comparisons (*α* = 0.05).

With respect to the gaze patterns, a cluster-based permutation analysis of looks to the Ongoing event picture (Fig 2, left panel) revealed a significant cluster of difference between the Imperfective and the Perfective condition stretching from 450 ms to 3000 ms after the Verb onset (sum *z* = −877.97, *p* < 0.001, represented by shading in Fig 3). This indicates that the participants looked at pictures of Ongoing events significantly more when they heard sentences containing an imperfective verb than when they heard sentences containing a perfective verb. The analysis of looks to the Completed event picture (Fig 2, right panel) also revealed a significant cluster of difference between the Imperfective and the Perfective condition stretching from 450 ms to 3000 ms after the Verb onset (sum *z* = 876.4, *p* < 0.001, represented by shading in Fig 2). This indicates that there were significantly more looks to pictures of Completed events when the target sentence contained a perfective verb than when it contained an imperfective verb.

### Upper estimate of effect latency

To investigate how quickly (relative to the verb onset) the participants were able to identify the target picture based on the aspectual information encoded in the verb we calculated the upper estimate of the effect latency. We identified 525 ms after the verb onset as the earliest time point where the probability of looks to the Target picture was significantly above chance after applying the Holm-Bonferroni correction for multiple comparisons, Fig 3. Given that the mean verb duration was 731 ms (range 441–982 ms), and that adults require approx. 200–250 ms to execute a saccade [73, 74], the upper estimate of the effect latency at 525 ms indicates that the participants were able to integrate the aspectual information already before they reached the end of the verb.

### Prefixal vs Suffixal aspectual marking

**Fig 4:**
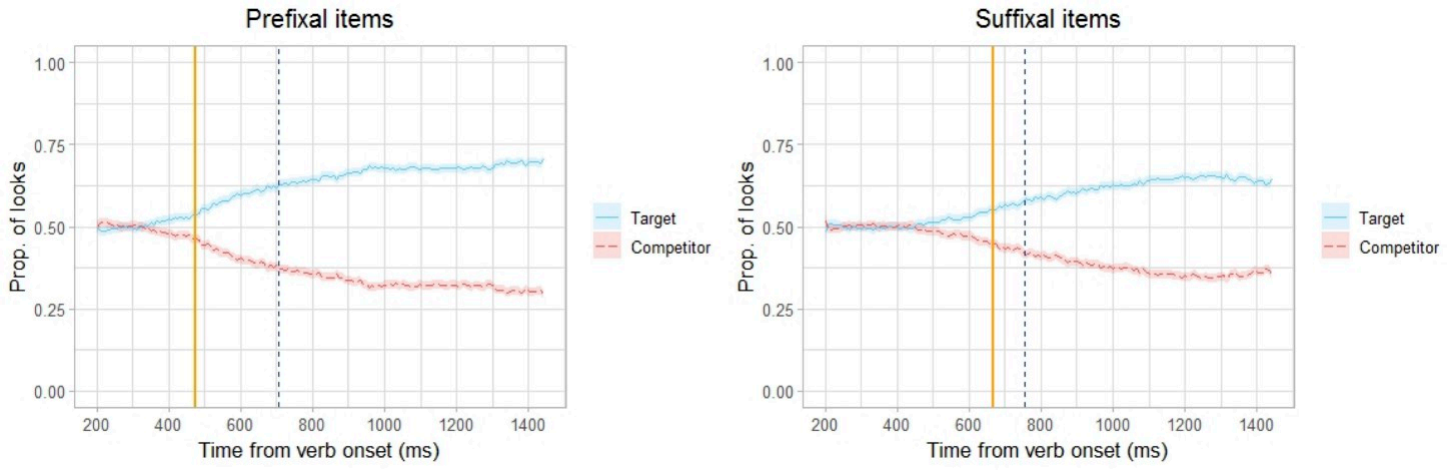
Prefixal vs Suffixal aspectual marking. Proportions of looks to the Target and Competitor pictures in Trials involving verbs with Prefixal and Suffixal aspectual marking starting from 200 ms after the verb onset. The vertical dashed blue lines represent the average verb offsets in the two conditions (706 ms for the Prefixal items and 755 ms for the Suffixal items). The vertical solid orange lines represent the estimates of effect latency (475 ms for the Prefixal items and 666 ms for the Suffixal items).

To compare the effect latencies between the Prefixal and Suffixal items we calculated latency estimates separately for the two conditions. The latency estimate, taken as the first of five consecutive time points with above-chance looks to the Target picture, was 475 ms for the Prefixal items and 666 ms for the Suffixal items, Δ = 192 ms (Fig 4).

To test whether the latency of the effect differed depending in the type of aspectual marking (Prefixal vs Suffixal) we conducted a BDP analysis. It generated bootstrapped distributions of the effect latency for the Prefixal items (mean = 491 ms, *SD* = 38.6), the Suffixal items (mean = 701 ms, *SD* = 64.6), and the difference in effect latencies between the Prefixal and Suffixal items (mean = 210 ms, *SD* = 75.1, see S3 Appendix). The 95% confidence interval for the difference in effect latencies was [58 ms, 358 ms]. The confidence interval does not include 0 which indicates that the difference in effect latencies between the Suffixal and Prefixal items was significant, i.e., the participants identified the Target picture significantly later when they heard verbs where the grammatical aspect distinction is expressed by the presence/absence of a suffix, as compared to verbs where this information is signalled by the presence/absence of a prefix.

This conclusion was confirmed by a BHT analysis which generated a bootstrap distribution of the difference in effect latency estimates under the null hypothesis that the type of aspectual marking (Prefixal vs Suffixal) does not have an effect (see S3 Appendix). The probability of the observed value (in our case, ≥ 192 ms) relative to the null hypothesis distribution was *p* = 0.008. Given than the *p*-value is lower than *α* = 0.05, we can reject the null hypothesis and conclude that the difference in effect latencies between the Suffixal and Prefixal items was significant. The bootstrap hypothesis testing procedure thus confirmed the results of the bootstrapping divergence point analysis, indicating that the processing of aspectual information was slower for verbs in Suffixal aspectual pairs than for verbs in Prefixal aspectual pairs.

The application of BDP and BHT analyses to the upper estimates of effect latencies for the Prefixal and Suffixal items yielded similar results. For the original data, these estimates were more conservative, especially in the Suffixal condition: 492 ms after the verb onset for the Prefixal items, and 758 ms for the Suffixal items, which amounts to a difference of 267 ms. The results of the analyses confirmed our conclusion that the difference in effect latencies between the Prefixal and Suffixal items was significant with [125 ms, 483 ms] as the 95% confidence interval for the difference in effect latencies and *p* = 0.005 as the probabillity of Δ = 267 ms relative to the null hypothesis distribution.

## Discussion

The participants’ gaze patterns revealed a strong and uniform preference for the completed event picture when they heard a sentence involving a perfective verb, and a strong and uniform preference for the ongoing event picture when they heard a sentence involving an imperfective verb. In terms of the time course of aspectual processing, the results showed that the participants were able to process and integrate grammatical aspect information incrementally on the sub-word level, already before they reached the end of the verb. Furthermore, the time course of processing was different depending on whether the aspectual information was available earlier or later in the verb. A comparison of eye-movements for the prefixal and suffixal verbal pairs revealed that aspectual information was integrated significantly earlier for the verbs in prefixal pairs than for the verbs in suffixal pairs. This further supports the conclusion that the participants were able to process and integrate grammatical aspect information in a highly incremental manner.

These results fit well with the cascaded model of language processing which assumes that all types of information (from the context and the linguistic stream) are integrated as soon as the information becomes available to the comprehender, and that integration can operate on incomplete or partial information [22, 25, 26]. With respect to our experiment, this entails a cascading, overlapping process whereby the integration of aspectual features with the information extracted from the visual context can begin already before the verb is fully presented to the listener. The onset of this process is correctly predicted to be tied to the exact location within the word where the aspectual information is encoded.

For the prefixal items, where aspectual information is disambiguated already at the onset of the verb, the preference for the Target picture was significantly above chance already at 475 ms after word onset. Subtracting 200–250 ms needed to plan and execute a saccade gives 225–275 ms as the probable time point where participants were able to process the aspectual information and integrate it with the visual context leading to preferential looks to the Target. This is compatible with the results of previous ERP studies of auditory speech processing which have identified 150–250 ms as the earliest time window when semantic incompatibility between a word and its preceding context is detected [22, 24, 26]. An interesting avenue for future research may involve a more detailed comparison between the time course of semantic processing as indexed by the relevant ERP components and the time course of target selection in Visual World eye-tracking experiments.

The proposed view of grammatical aspect processing makes specific predictions about the latency of the processing effect depending on the type of aspectual marking a language employs. For instance, in some languages aspectual meaning can be expressed by analytic constructions with an auxiliary verb preceding the lexical verb (e.g., the progressive and perfect in English and Italian, the perfect in Dutch and Norwegian, etc.). Given our results, we expect that comprehenders would be able to make rapid use of the information encoded in the auxiliary and the initial segments of the lexical verb. In other languages aspectual categories are encoded as part of the inflection at the very end of the verb, e.g., in the Preterite (past perfective) and Imperfect (past imperfective) tense forms in Spanish, or in post-verbal aspectual particles (e.g., in Mandarin Chinese). In this case we predict that the identification of the target picture in a Visual World setup will be delayed until after the end of the verb. This is confirmed by the results of a previous Visual World eye-tracking study of aspect processing in Mandarin Chinese [46], which showed that the identification of the target picture occurs shortly after the presentation of the post-verbal aspectual particle.

We should point out that a certain degree of caution is warranted with regards to the broader implications of our results. Our experiment employed the Visual World paradigm, where the audio linguistic input was combined with a strong visual context. Our results show that Russian speakers were able to integrate aspectual information in a highly incremental manner, however further research is needed to evaluate the extent to which the type of context presented to the listeners can have an effect on the incremental processing of aspect.

We should also note that this study focused specifically on verbal pairs where the Imperfective verb denotes an activity while the Perfective verb refers to the natural culmination of that activity. We considered two types of such pairs: prefixal pairs where the aspectual distinction is marked by the presence/absence of a prefix and suffixal pairs where it is marked by the presence/absence of a suffix. Interestingly, in Russian the same morphological means (perfectivizing prefixes and imperfectivizing suffix) can also be used to convey other semantic contrasts, e.g.:

a) activity/state vs beginning of activity/state: *p^j^e-* (IMP) ‘sing’ vs *za-p^j^e-* (PFV) ‘start singing’, *os̆^j^us̆^j^a-* (IMP) ‘feel’ vs *os̆^j^uti-* (PFV) ‘start feeling’;
b) activity vs temporally bounded activity: *p^j^e-* (IMP) ‘sing’ vs *po-p^j^e-* (PFV) ‘sing for a while’;
c) motion vs spatially bounded motion: *b^j^ez̆a-* (IMP) ‘run’ vs *v-b^j^ez̆a-* (PFV) ‘run inside’; etc.

Based on the results of the current study, we would predict rapid incremental processing of aspectual marking in such pairs as well. Further research is needed to determine if this prediction is correct or if the type of semantic opposition conveyed by aspectual morphology has an impact on the time course of verb processing.

The predicate pairs included in this study can also be divided into various sub-types, e.g., depending on whether the nature of the culmination state is inherent in the semantics of the Imperfective verb (e.g., *v^j^es̆at^j^/pov^j^esit^j^ kartinu* ‘to hang a painting’) or derived from the bounded nature of the theme argument (e.g., *krasit^j^/pokrasit^j^ stenu* ‘to paint a wall’). Similarly the pairs can be differentiated based on whether the prefix on the Perfective verb possesses a more abstract meaning (e.g., *po-* in *po-stroit^j^* ‘to build’, *po-c̆init^j^* ‘to fix’, *po-myt^j^* ‘to wash’, etc.) or can be associated with a more specific semantic content (e.g., *pro-* in *pro-sv^j^erlit^j^* ‘to drill’ can be linked with the semantics of moving *through* something; *raz-* in *raz-r^j^ezat^j^* ‘to cut’ is used to convey the notion of *division* of something into parts). The data obtained in our study does not allow for a systematic comparison between these sub-types of verbal pairs. Additional experiments specifically designed to control for these factors need to be conducted in order to determine if they have a discernible effect on processing.

A hotly debated question in the study of Russian aspect, partially relevant for our study, is what constitutes an *aspectual pair*. Aspectual pairs are traditionally defined as pairs of Imperfective and Perfective verbs where the Perfective verb can be substituted by the Imperfective one in contexts that do not allow the use of Perfective aspect (e.g., in habitual and Historical Present contexts, [37, 40]). It is commonly assumed that suffixal pairs (i.e., pairs where the Imperfective verb is derived from the Perfective by the addition of a suffix) constitute true aspectual pairs. Most researchers believe that aspectual pairs can also be prefixal, i.e., can be formed by deriving a Perfective verb from an Imperfective verb the help of a perfectivizing prefix [39, 40, 75]. The latter assumption, however, is not universally accepted. For instance, Isačenko [76] argues that prefixal verb pairs are never true aspectual pairs because the prefix on the Perfective verb necessarily introduces a meaning component that is not present in the interpretation of the non-prefixed Imperfective verb. Consequently, Imperfective verbs in such pairs can never replace Perfective verbs without a change in meaning. Now, the selection criterion for the items in our study (both in prefixal and suffixal pairs) was not based on substitutivity, but on the semantic properties of the verbs: the members of each pair refer to the same type of event, with the Imperfective verb denoting an activity and the Perfective verb referring to the natural culmination of that activity. Thus, the issue of what constitutes a ‘true aspectual pair’ is at least partly orthogonal to our study (see the relevant discussion of the distinction between *actional pairs* and *aspectual pairs* in [36]). Moreover, the semantic parallelism between prefixal and suffixal pairs is supported by the results of Janda and Lyashevsakaya’s [77] wide-scale corpus study. They found that the distributions of the verbal forms in the Russian National Corpus (92 million words) were the same regardless of the type of aspectual marking, which they interpreted as evidence against Isac̆enko’s position. Consequently, we believe that the difference in processing latency that we observed between the prefixal and suffixal items in our study is most likely explained by the difference in where within the verb the aspectual information is encoded (i.e., the location of the disambiguation point), rather than some systematic semantic difference between the two types of pairs.

Finally, from a methodological point of view, our study has demonstrated how a range of analytical techniques can be successfully applied to investigate the fine-grained time course of linguistic processing within the Visual World eye-tracking paradigm. In particular, we have shown how bootstrapping divergence point analysis can be used to detect subtle, but significant differences in effect latencies on a sub-word scale (see [66], for an application of this method to cross-group comparison in Visual World eye-tracking). We believe that this method can be fruitfully employed in the investigation of fine-grained incremental processing in a wide range of eye-tracking studies.

## Supporting information

S1 Appendix Test items. (PDF)

S2 Appendix Filler items. (PDF)

S3 Appendix BDP and BHT distributions. (PDF)
