# Supplementary material for: Fine-grained time course of verb aspect processing
Source: PLoS One. 2022 Feb 25;17(2):e0264132. doi: 10.1371/journal.pone.0264132 (PMC8880397; doi:10.1371/journal.pone.0264132)
Supplement: S1 Appendix — (PDF) [file pone.0264132.s001.pdf]

Preamble	Target sentence	Aspectual marking
Deti igrali v komnat^{j}e.<br>‘The children were playing in the room.’	Malčik **stroil/postroil** vysokuju bašn^{j}u.<br>‘The boy was building/built a tall tower.’	Prefixal
Byl prazdnik.<br>‘It was a holiday.’	D^{j}eduška **gladil/pogladil** novuju rubašku.<br>‘Grandpa was ironing/ironed a new shirt.’	Prefixal
Byl solnečnyj d^{j}en^j.<br>‘It was a sunny day.’	Babuška **sažala/posadila** b^{j}elyij cv^{j}etok.<br>‘Grandma was planting/planted a white flower.’	Prefixal
Šël pervyj urok.<br>‘It was the first lesson.’	Malčik **točil/potočil** tonkij karandaš.<br>‘The boy was sharpening/sharpened a thin pencil.’	Prefixal
Bylo zimneje utro.<br>‘It was a winter morning.’	Babuška **v^{j}azala/sv^{j}azala** novyj sviter.<br>‘Grandma was knitting/knitted a new sweater.’	Prefixal
Byl vyxodnoj d^{j}en^j.<br>‘It was the weekend.’	D^{j}eduška **činil/počinil staryj** xolodil^{j}nik.<br>‘Grandpa was fixing/fixed an old fridge.’	Prefixal
Byl vyxodnoj d^{j}en^j.<br>‘It was the weekend.’	Babuška **v^{j}ešala/povesila** kraisvuju kartinu.<br>‘Grandma was hanging up/hung up a beautiful painting.’	Prefixal
Šël p^{j}ervyj urok.<br>‘It was the first lesson.’	D^{j}evočka **risovala/narisovala** tonkuju vazu.<br>‘The girl was drawing/drew a thin vase.’	Prefixal
Sem^{j}ja d^{j}elala r^{j}emont.<br>‘The family was doing repairs.’	D^{j}evočka **krasila/pokrasila** vysokuju st^{j}enu.<br>‘The girl was painting/painted a tall wall.’	Prefixal
Bylo vr^{j}em^{j}a ob^{j}eda.<br>‘It was lunchtime.’	Malčik **myl/pomyl** beluju tarelku.<br>‘The boy was washing/washed a white plate.’	Prefixal
Sem^{j}ja d^{j}elala r^{j}emont.<br>‘The family was doing repairs.’	D^{j}eduška **sv^{j}erlil/prosv^{j}erlil** bol^{j}šuju dyrku.<br>‘Grandpa was drilling/drilled a big hole.’	Prefixal
Bylo zimneje utro.<br>‘It was a winter morning.’	D^{j}eduška **l^{j}epil/sl^{j}epil** bol^{j}šogo sn^{j}egovika.<br>‘Grandpa was building/built a big snowman.’	Prefixal
Byl prazdnik.<br>‘It was a holiday.’	D^{j}evočka **naduvala/nadula** zeljënyj šarik.<br>‘The girl was blowing up/blew up a green balloon.’	Suffixal
Byl solnečnyj d^{j}en^j.<br>‘It was a sunny day.’	D^{j}eduška **vykapyval/vykopal** glubokuju jamu.<br>‘Grandpa was digging/dug a deep pit.’	Suffixal
Byl pold^{j}en^j.<br>‘It was noon.’	Malčik **podm^{j}etal/podm^{j}ël** dlinnyj koridor.<br>‘The boy was sweeping/swept a long corridor.’	Suffixal
Bylo vr^{j}em^{j}a ob^{j}eda.<br>‘It was lunchtime.’	Babuška **razr^{j}ezála/razr^{j}ézala** zel^{j}ënyj arbuz.<br>‘Grandma was slicing/sliced a green watermelon.’	Suffixal
Deti igrali v komnat^{j}e.<br>‘The children were playing in the room.’	D^{j}evočka **sobirala/sobrala** krasivyj zamok.<br>‘The girl was constructing/constructed a beautiful castle.’	Suffixal
Bylo zimneje utro.<br>‘It was a winter morning.’	D^{j}eduška **razžigal/razžëg** staryj kamin.<br>‘Grandpa was lighting/lit an old fireplace.’	Suffixal
Stojala xorošaja pogoda.<br>‘The weather was good.’	D^{j}evočka **pereplyvala/preplyla** glubokuju r^{j}eku.<br>‘The girl was swimming/swam across a deep river.’	Suffixal
Bylo voskr^{j}es^{j}en^{j}je.<br>‘It was Sunday.’	Malčik **ubiral/ubral** bol^{j}šuju komnatu.<br>‘The boy was tidying up/tidied up a large room.’	Suffixal
Deti igrali v komnat^{j}e.<br>‘The children were playing in the room.’	Malčik **raskrašival/raskrasil** krasivyj risunok.<br>‘The boy was coloring in/colored in a pretty picture.’	Suffixal
Stojala xorošaja pogoda.<br>‘The weather was good.’	Babuška **polivala/polila** zel^{j}ënyj kust.<br>‘Grandma was watering/watered a green bush.’	Suffixal
Byl pold^{j}en^j.<br>‘It was noon.’	Babuška **zapirala/zaperla** beluju dv^{j}er^j.<br>‘Grandma was locking/locked a white door.’	Suffixal
Byl prazdnik.<br>‘It was a holiday.’	D^{j}evočka **otkryvala/otkryla** krazivyj podarok.<br>‘The girl was opening/opened a beautiful present.’	Suffixal
