# Supplementary material for: Fine-grained time course of verb aspect processing
Source: PLoS One. 2022 Feb 25;17(2):e0264132. doi: 10.1371/journal.pone.0264132 (PMC8880397; doi:10.1371/journal.pone.0264132)
Supplement: S2 Appendix — (PDF) [file pone.0264132.s002.pdf]

| Preamble | Target sentence |
|---|---|
| Bylo voskrʲesʲenʲje.<br>‘It was Sunday.’ | Babuška ispʲekla boljšoj pirog. *<br>‘Grandma baked a big cake.’ |
| Šël pervyj urok.<br>‘It was the first lesson.’ | Dʲevočka vyrjezála krasivyj risunok. *<br>‘The girl was cutting out a pretty picture.’ |
| Semʲja dʲelala rʲemont.<br>‘The family was doing repairs.’ | Malčik zabil dlinnyj gvozʲdʲ. *<br>‘The boy hammered in a long nail.’ |
| Bylo xolodno.<br>‘It was cold.’ | Dʲevočka sžigala sinij žurnal. *<br>‘The girl was burning a blue magazine.’ |
| Byl večer.<br>‘It was evening.’ | Dʲevočka vypila beloje moloko. *<br>‘The girl drank the white milk.’ |
| Stojala doždlivaja pogoda.<br>‘It was rainy.’ | Babuška šila novoje platʲje. *<br>Grandma was sewing a new dress. |
| Byla tʲëmnaja noč.<br>‘It was a dark night.’ | Malčik zakopal dʲerʲevʲannyj sunduk. *<br>‘The boy buried a wooden chest.’ |
| Bylo xolodno.<br>‘It was cold.’ | Dʲeduška razrušal čërnyj dom. *<br>‘Grandpa was demolishing a black house.’ |
| Byl solnečnyj dʲenʲ.<br>‘It was a sunny day.’ | Dʲeduška srubil zelʲënoje dʲerʲevo. *<br>‘Grandpa chopped down a green tree.’ |
| Byl solnečnyj dʲenʲ.<br>‘It was a sunny day.’ | Malčik pilil dlinnoje brʲevno. *<br>‘The boy was sawing a long log.’ |
| Byl večer.<br>‘It was evening.’ | Dʲevočka sjela krasnuju rybu. *<br>‘The girl ate a red fish.’ |
| Byla tʲëmnaja noč.<br>‘It was a dark night.’ | Dʲeduška potušil staruju svʲeču.<br>‘Grandpa blew out an old candle.’ |
| Byl solnečnyj dʲenʲ.<br>‘It was a sunny day.’ | Malčik fotografiroval sʲeruju goru.<br>‘The boy was photographing a grey mountain.’ |
| Byl vyxodnoj dʲenʲ.<br>‘It was the weekend.’ | Babuška zamorozila sladkij sok.<br>‘Grandma froze some sweet juice.’ |
| Bylo voskrʲesʲenʲje.<br>‘It was Sunday.’ | Malčik napisal dlinnoje pisʲmo.<br>‘The boy wrote a long letter.’ |
| Byl vyxodnoj dʲenʲ.<br>‘It was the weekend.’ | Dʲeduška gotovil novyj dʲesʲert.<br>‘Grandpa was preparing a new desert.’ |
| Byl poldʲenʲ.<br>‘It was noon.’ | Babuška otrʲézala dyrʲavyj rukav.<br>‘Grandma cut off a torn sleeve.’ |
| Byl poldʲenʲ.<br>‘It was noon.’ | Malčik razbiral bʲeluju taburʲetku.<br>‘The boy was disassembling a white stool.’ |
| Stojala doždlivaja pogoda.<br>‘It was rainy.’ | Dʲeduška čital staruju knigu.<br>‘Grandpa was reading an old book.’ |
| Bylo rannʲeje utro.<br>‘It was early in the morning.’ | Dʲevočka podoila bolʲšuju korovu.<br>‘The girl milked a big cow.’ |
| Byl solnečnyj dʲenʲ.<br>‘It was a sunny day.’ | Dʲeduška polol bolʲšuju grʲadku.<br>‘Grandpa was weeding a big strawberry patch.’ |
| Byla tʲëmnaja noč.<br>‘It was a dark night.’ | Dʲeduška vzorval dlinnyj most.<br>‘Grandpa blew up a long bridge.’ |
| Bylo rannʲeje utro.<br>‘It was early in the morning.’ | Dʲevočka kupila novyj tʲelʲefon.<br>‘The girl bought a new phone.’ |
| Byl vyxodnoj dʲenʲ.<br>‘It was the weekend.’ | Babuška kolola krglyj orʲex.<br>‘Grandma was cracking a round nut.’ |
