# Supplementary material for: Fine-grained time course of verb aspect processing
Source: PLoS One. 2022 Feb 25;17(2):e0264132. doi: 10.1371/journal.pone.0264132 (PMC8880397; doi:10.1371/journal.pone.0264132)
Supplement: S3 Appendix — (PDF) [file pone.0264132.s003.pdf]

S3 Appendix. BDP and BHT distributions.

Bootstrap divergence point analysis (BDP)

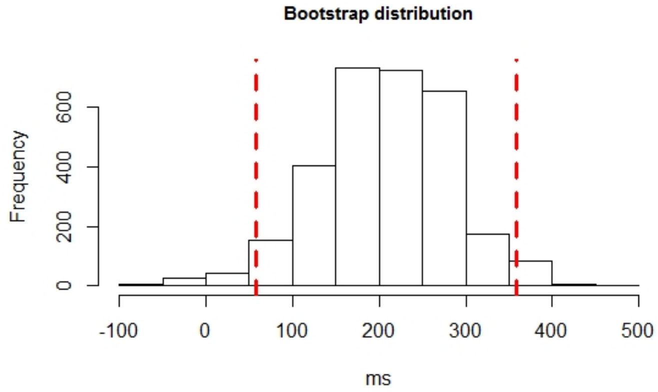

Fig S1. Bootstrap distribution of the difference in effect latency estimates for the Prefixal and Suffixal items.
The vertical dashed red lines mark the boundaries of the 95% confidence intervals (Percentile method: [58 ms, 358 ms]).

Bootstrap hypothesis testing (BHT)

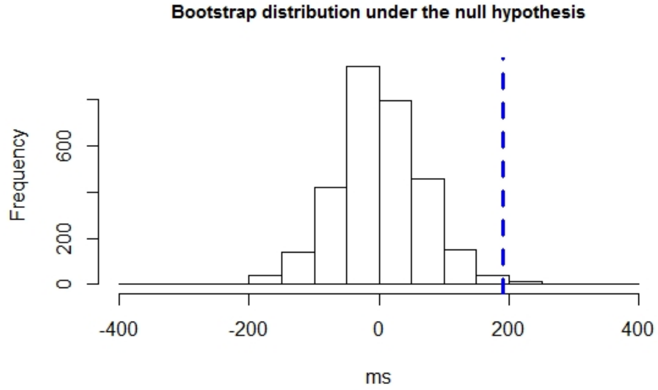

Fig S2. Bootstrap distribution of the difference in effect latency estimates for the Prefixal and Suffixal items under the null hypothesis.
The vertical dashed blue line represents the actual difference in latency estimates (192 ms, $p = 0.008^{**}$).
